# Leading the way toward a socially-accountable medical education system

**Published:** 2019-03-13

**Authors:** Justin Hall, Reza Mirza, Pavandeep Gill

**Affiliations:** 1 Division of Emergency Medicine, Department of Medicine, University of Toronto, Ontario, Canada; 2 Resident Doctors of Canada, Ontario, Canada; 3 Division of Internal Medicine, Department of Medicine, McMaster University, Ontario, Canada; 4 Department of Pathology & Laboratory Medicine, University of Calgary, Alberta, Canada

Fostering a healthy population and a high quality, efficient healthcare system necessitates socially-accountable, purposeful use of resources and active collaboration among all healthcare providers and communities we serve. As we develop our professional identity during our transitions from medical students to resident doctors to independent, practicing physicians, we increasingly understand our essential role as healthcare system stewards. Through our journey, however, we have discovered that our medical education was not designed to support us in becoming health-system leaders. Each level of our medical education is a silo of governance, assessment, examination, and accreditation. While there are institutions and programs that excel at instilling social accountability in their graduates in a meaningful way, these important principles can certainly be expanded to become a more robust component of medical education.

The inclusion of social accountability in medical education is the result of concerted effort from multiple stakeholders. An early driver in prioritizing social accountability was the World Health Organization’s 1995 report which defined the social accountability mandate of medical schools as “the obligation to direct their education, research and service activities towards addressing the priority health concerns of the community, region, and/or nation they have a mandate to serve.”^1^ This mandate was further articulated with the Charter of Medical Professionalism. Developed by representatives of North American and European medical associations in 2002, the Charter describes the social contract between the medical profession and society built on the foundational trust that the public has placed in physicians. It details medical schools’ accountability to train physicians to meet the needs of the population.^2^ Moreover, the Accreditation Council for Graduate Medical Education (ACGME) accreditation competencies, CanMEDS (Canadian Medical Education Directions for Specialists) 2015 framework for medical education, and CanMEDS-FM framework for family medicine training reinforce the position that social accountability, leadership, and resource stewardship are essential competencies for all physicians in training.^3^^-5^ One example illustrating the need for these competencies stems from studies demonstrating that clinician ordering patterns are influenced by their residency training, and that these patterns persist as staff physicians.^6^^-^^8^

While social accountability has permeated our medical schools, tensions remain regarding the relative importance of different elements of a medical school’s mission. Research and education have traditionally superseded social accountability for many institutions with difficulty in measuring social accountability often cited as a barrier to achieving the social contract.^9^^,^^10^ Moreover, medical education has sometimes been viewed as a process to develop medically-expert physicians with less emphasis on creating socially-accountable physicians who serve their communities and effect change to reduce health disparities.^11^ We strive to embody and embrace the duality of clinical excellence and health stewardship as necessary attributes for all doctors.

As we navigate these transitions, adopt new roles and responsibilities, and meet new expectations, we must cultivate more comprehensive skill sets. These skills include leadership, advocacy, practice management, resource stewardship, and building organizational partnerships, among others. Resident Doctors of Canada (RDoC) is exploring ways to ease these challenging transitions and optimize success for all residents.

At the organizational level, by explicitly considering equity, transparency, sustainability, and relevance across all initiatives and policy directions, RDoC is leading the way toward creating a socially-accountable healthcare system.^12^ To illustrate, RDoC has advocated over the last several years for a pan-Canadian strategy that manages and aligns physician health human resources (HHR) with population needs as an essential component of a publicly-funded high-quality healthcare system. Many Canadian residency graduates, from a range of specialties and programs across the country, have trouble securing full-time meaningful employment, which not only creates unnecessary anxiety for residents, but also has a significant impact on Canadian patients and their access to healthcare services.^13^ RDoC partners with the Canadian Federation of Medical Students and supports their work with medical schools to shape a physician workforce that is diverse across dimensions including gender, ethnicity, and socioeconomic status to better meet the needs of the diverse Canadian population. Moreover, RDoC has developed a series of resident doctor profiles as a means of creating awareness about different specialties, helping prepare residents to work more effectively in multidisciplinary, inter- and intra-professional teams to enhance patient care. RDoC remains a strong voice for effective, evidence-based workforce planning and partners with governments, hospitals, and other organizations to build value, improve efficiency, and achieve effectiveness in the distribution and utilization of healthcare resources.

Another example involves expanded practice management training. RDoC is working to advance the standardization of a practice management curriculum across all Canadian residency programs to better prepare residents for the non-clinical aspects of running a practice. This includes training in the legal, administrative, and financial aspects of medicine to ensure residents develop the necessary skills to practice within their specialities. In addition, RDoC is working with the Canadian Medical Protective Association to design and deliver interactive educational sessions, for residents across all Canadian faculties, in the areas of patient safety and medicolegal risk reduction. For each of these initiatives, RDoC engages in a process of continuous quality improvement and outcome evaluation aimed at ensuring we are serving the public optimally and training physicians to meet the healthcare needs of all Canadians.

Healthcare utilization practice is another key concept to consider in the social accountability of doctors in Canada, the United States, and beyond. Today, up to 30% of tests and treatments that physicians order may be unnecessary and may cause harm.^14^^-^^16^ To address this misalignment, RDoC advocates with our partner organizations for a medical education system that sees social accountability as a fundamental foundation, and promotes resource stewardship awareness and evidence-based care to identify and reduce unnecessary care in our own practices.^17^ This is an essential component of a socially-accountable, patient-centered healthcare system. Potential future initiatives include enhancing educational transition supports, building further leadership capacity, advocating for social accountability curricula within postgraduate training programs, supporting trainee-led work on resource stewardship, and promoting resource stewardship collaboration across health disciplines. To optimize the likelihood of successful change and system impact, these efforts will need to be coordinated and integrated within the larger tapestry of health care training and delivery at undergraduate, postgraduate, and continuing professional development levels. As we continue our training and transition toward independent practice, we strive to build an evolving and sustainable healthcare system that sees social accountability as the lens through which we become leaders in education, research, administration, and care delivery.
